# Le lambeau bilobe de Zitelli dans la reconstruction d’un CBC de la pyramide nasale

**DOI:** 10.11604/pamj.2017.27.238.11135

**Published:** 2017-08-02

**Authors:** Karim Bourra, Samir Mazouz

**Affiliations:** 1 Service Chirurgie Plastique, Brûlés esthétiques, Hôpital al Farabi, Oujda, Maroc; 2 Service Chirurgie Plastique, Brûlés et Chirurgie de la Main, Hôpital Avicenne, Rabat, Maroc

**Keywords:** Carcinome basocellulaire, carcinome spino-cellulaie, kératoacanthome, lambeau local, Basal cell carcinoma, spinocellular carcinoma, keratoacanthoma, local flap

## Abstract

We report the case of a 65-year old patient presenting with well-defined nodular basal cell carcinom measuring 5mm in diameter in the right paramedian nasal tip and the superior margin of the nasal wing, near the free edge. The tumor was removed, with resection margin of 1mm. It was associated with multiple hyperkeratotic lesions spread across his face. The patient was a farmer with a past history of massive sun exposure. He lived in rural, mountainous and remote regions. The nasal tumor had evolved very slowly and very gradually in volume. The patient underwent surgical excision of BCC with resection margin of 1mm. We reconstructed the loss-of-substance with a very simple small local flap (Zitelli bilobed flap) which allowed to cover the loss of substance and to close it. The recipient healed well after one month-follow-up of local treatment and dressings. The patient underwent the removal of the sutures on day 10, then he underwent fat dressings with cortisone cream every other day over a period of 15 days.

## Image en médecine

Homme de 65 ans présente un carcinome basocellulaire nodulaire, bien limité de 5mm de diamètre sur la pointe nasale paramédiane droite et le sommet de l'aile narinaire, à proximité du bord libre. La tumeur est retirée avec des marges latérales de sécurité de 1mm. Elle est associée à des lésions hyperkératosiques multiples dispersées un peu partout au visage. Il y a une notion d'exposition solaire massive vu que notre patient est un paysan et qui réside dans des régions montagneuses rurales éloignées. La tumeur nasale a augmenté très lentement et très progressivement de volume. Nous avons procédé à l'exérèse chirurgicale du CBC avec des marges de 1mm. Nous avons reconstruit la perte de substance laissée sur place par un petit lambeau local très simple appelé « Lambeau de ZITELI », qui a permis de couvrir la perte de substance et de la fermer. La zone receveuse a bien cicatrisé au bout d'un mois de suivi par traitement local et des pansements. L'ablation de fils de sutures est faite à J10, puis des pansements gras et cortisonés sont mis un jour sur deux pendant 15 jours.

**Figure 1:**
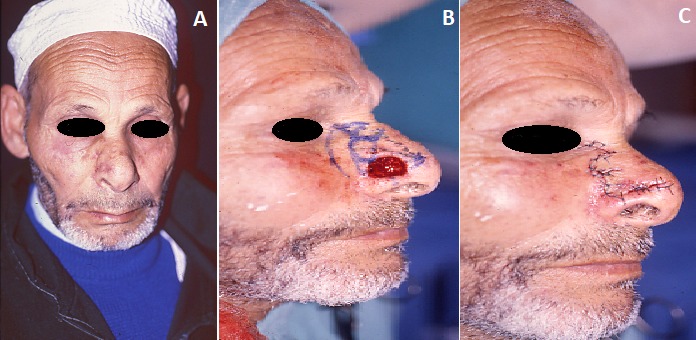
(A) CBC de la face droite de la pyramide nasale; (B) résection du CBC avec des marges de 1mm et dessin du lambeau; (C) lambeau de ZITELI mis en place et sutures cutanées par points simples

